# Computational–Experimental Identification of Palindromic Motifs Bound by Bacterial XRE Family Transcriptional Regulators

**DOI:** 10.3390/life15101577

**Published:** 2025-10-10

**Authors:** Linjia Wang, Shitong Zhong, Liangyan Wang, Huizhi Lu, Yuejin Hua

**Affiliations:** 1 MOE Key Laboratory of Biosystems Homeostasis & Protection, Institute of Biophysics, College of Life Sciences, Zhejiang University, Hangzhou 310058, China; linjia1232021@163.com (L.W.); zhongshitong@zju.edu.cn (S.Z.); liangyanwang@zju.edu.cn (L.W.); 2 Cancer Center, Zhejiang University, Hangzhou 310058, China

**Keywords:** palindromic motif, XRE family, transcriptional regulators, motif clustering, AlphaFold, protein–DNA interactions

## Abstract

Bacteria employ transcriptional regulators, such as those belonging to the Xenobiotic Response Element (XRE) family, to regulate metabolic processes. These regulators often exhibit autoregulatory properties and function as dimers to recognize palindromic DNA motifs. However, the binding motifs of the XRE family transcriptional regulators in bacteria have not yet been well characterized on a large scale. To identify potential XRE transcriptional regulator recognition motifs efficiently, we developed a computational approach combining structural alignment, sequence scanning, and motif clustering. We first identified the potential motifs of XRE regulators using computational methods. Using the helix–turn–helix (HTH) domain of XRE family regulators as a template, we collected 27,732 proteins containing the domain from bacterial databases. By extracting upstream sequences of these proteins and employing bioinformatics tools like MEME and motifStack to search potential motifs, 5622 motifs were identified and subsequently clustered into 223 clusters. These clusters can be classified into 7 main types based on the base conservation patterns observed in motifs. Interaction models between representative proteins and their corresponding motifs were predicted using AlphaFold. Subsequently, we conducted experimental validation via electrophoretic mobility shift assays (EMSAs) and confirmed the feasibility of our approach, as nine out of ten tested interactions showed clear protein–DNA binding. However, due to limitations in experimental conditions, the remaining predicted motifs have not yet undergone experimental validation. Since conserved sequences and well-predicted structures cannot replace real-world scenarios, there are limitations to relying solely on computational predictions, and experimental validation remains necessary. In summary, our study establishes a reliable framework for identifying XRE family transcriptional regulator recognition motifs and provides valuable insights into bacterial regulation.

## 1. Introduction

Bacteria rely on various transcriptional regulators to control metabolic processes during their life activities. These transcriptional regulators are crucial for modulating gene expression in response to internal and external stimuli, enabling bacteria to adapt rapidly to changing environments. Over the past decades, several major families of bacterial transcription regulators have been extensively studied, including LysR [1], TetR [2], Arac/XylS [3], MerR [4,5], GntR [6,7], and XRE families [8]. Each of these families exhibits distinct structural features and regulatory mechanisms.

Among the known families of transcriptional regulators, the Xenobiotic Response Element (XRE) family is highly prevalent in bacteria and represents the second most common family of regulators involved in diverse metabolic functions [8,9]. XRE regulators typically function by binding to the promoter regions of target genes through their HTH domain [10,11], functioning as transcriptional repressors or activators [10,11,12]. Beyond their basic DNA-binding functions, some also sense environmental signals; for example, MsrR in *Corynebacterium glutamicum* senses oxidative stress through a thiol-based mechanism, modulating its DNA-binding capacity to respond to redox changes [13]. Similarly, PsdR in *Pseudomonas aeruginosa* contains a copper protein signaling sensor domain and functions as a quorum-sensing regulator [14].

Overall, XRE family regulators play a significant role in diverse cellular processes, including antibiotic metabolism [15,16,17], stress and damage responses [12,13,18,19,20] and phenotypic switching [11]. For example, PsdR and PauR in *Pseudomonas aeruginosa* control dipeptide and polyamine metabolism, conferring growth advantages under environmental challenges [15]; SrtR in *Streptococcus suis* regulates oxidative stress tolerance and strain virulence [20]; DNA damage-induced protein A (DdiA) in *Myxococcus xanthus* regulates LexA-independent DNA damage response (DDR) pathways [18]; and XtrSs in *Streptococcus suis* binds palindromic promoter sequences to autoregulate its transcription and oxidative stress resistance [19]. Moreover, XRE regulators encoded by bacteria and bacteriophages, such as the shared cluster in *Caulobacter crescentus* and its phage φCbK, strongly influence host–phage dynamics and adhesion regulation [21], highlighting their central role in bacterial adaptation and survival. The XRE regulators can be found in multiple pathways within bacteria, which underscores their importance in bacterial populations.

Notably, most XRE family regulators exhibit autoregulatory properties, in which they are able to bind to the upstream sequences of their own open reading frames, such as ImmR [22,23], DdrO [12], SCO1979 [16], MsrR [13], and PrpR [24]. Additionally, many of these regulators function as dimers, for instance, ImmR [25], DdrO [12], and MltR [26] form dimers and cooperate with palindromic promoter DNA, which implies that the motifs they recognize could be palindromic.

Traditionally, identifying transcription regulator binding sites often relies on experimental approaches such as ChIP-seq or sequence truncations methods [8,27]. Traditional experimental methods are highly accurate and effective for identifying the motifs of individual regulators in specific species. However, when it comes to large-scale motif identification, experimental methods are difficult to advance due to the explosive workload and high experimental costs. Therefore, for large-scale motif screening, experimental methods are relatively inefficient and far from ideal, so computational methods ought to be taken into account.

For XRE regulators, the identification of well-defined binding motifs is lacking. Currently, a few regulators such as DdrO [28] and XRE-cupin TFs [15] have clearly identified motifs. However, for many other regulators [9,10,11,18,20], only a specific segment of DNA sequence known to be bound can be identified, with no summarized motifs available. Yet the summary and identification of motifs are crucial for studying the mechanism of regulators; therefore, based on these two features that most XRE family regulators exhibit autoregulatory and dimerization properties, we aim to collect the upstream sequences of homologous XRE family transcription factors, seeking out any palindromic sequences within them, and experimentally validate the accuracy of the identified sequences.

RegPrecise Database (regprecise.lbl.gov) [29] explicitly annotates that XRE family regulators share a characteristic N-terminal HTH DNA binding domain (PF01381), while their C-terminal regulatory region is highly variable. Furthermore, in the Conserved Domain Database (CDD) [30], the XRE family corresponds to COG1476 (XRE-family HTH domain), which served as our key structural classification criterion. The C-terminal regulatory region of XRE family regulators demonstrates substantial diversity, leading to functional variability. These regulators play integral roles in a wide array of biological processes and exhibit profound effects on cellular functions. Therefore, the palindromic motif analysis of XRE family transcriptional regulators is of great significance, and our research aims to develop a scalable computational approach to identify and classify palindromic motifs of bacterial XRE regulators and experimentally validate the predictions. In this study, we utilized the HTH domain of XRE family regulators to collect a large number of protein sequences from bacterial protein databases via structural alignment. By employing Basic Local Alignment Search Tool (BLAST, version 2.12.0) and sequence scanning techniques, we systematically identified and excavated potential palindromic sequences within these proteins. Subsequently, we conducted motif clustering analysis to pinpoint high-confidence motif types. Furthermore, we experimentally validated the interaction between selected motifs and the corresponding proteins, thereby demonstrating the effectiveness of our screening approach.

## 2. Materials and Methods

### 2.1. The Collection of XRE Family Proteins

First, the N-terminal domain (XRE domain) of the predicted structure of DR_2574 was extracted. Using the FoldSeek server [31] with default parameters (the databases selected were AlphaFold/UniProt50 v4, and the mode was set to 3Di/AA), homologous proteins containing the same XRE domain were identified within the bacterial kingdom (database: AlphaFold/Uniprot50), resulting in the collection of 1000 protein entries. Subsequently, the last protein containing XRE domain from collection was selected (this choice favors the expansion of the protein set while preserving structural similarity), and its XRE domain was extracted to repeat the aforementioned collection process. After removing redundant proteins, the process continued by selecting proteins containing the XRE domain that were positioned further downstream, extracting their XRE domains, and repeating the collection procedure. This iterative process was repeated 100 times, thereby completing the preliminary collection of XRE family proteins.

### 2.2. The Construction of the BLAST Database

First, all reference genomes of bacterial taxa were downloaded from NCBI (downloaded on 10 December 2024). Protein sequences were then extracted from these genomes and consolidated into a single FASTA file. Subsequently, the makeblastdb tool (version 2.12.0) was used to construct a local BLAST database from the FASTA file.

### 2.3. Motif Discovery and Clustering

The specific code for motif discovery and clustering steps can be accessed on GitHub (https://github.com/Zpresitong/XRE-motif.git, accessed on 3 July 2025).

For each collected XRE family protein, a local BLAST search was conducted. For each BLAST search, up to 120 protein entries were retained, and the DNA sequence upstream of each protein’s reading frame (200 bp, selected to encompass the promoter region) was extracted from the genome file and recorded in a FASTA-formatted file. Subsequently, the MEME suite (version 5.5.7) was used for motif discovery based on the DNA sequence information (command: “meme promoter_seqs.txt -dna -minw 15 -maxw 25 -mod anr -pal -nmotifs 3 -brief 1000 -nostatus -p 1”). Taking the 17-bp-wide motif of DdrO [28] as an example, the minimum and maximum motif widths set in the parameters are considered sufficient to cover most potential motifs.

After completing all BLAST searches and motif discoveries, the collected motifs were subjected to preliminary screening. Motifs with fewer than 50 matching proteins, sequence coverage below 75%, or non-conserved sequences were discarded. The remaining motif information was clustered using the motifStack package in R, and a clustering tree was constructed. Based on branch distances, the motifs were initially classified, and any categories with fewer than five branches were removed.

A shared motif analysis was performed on the preliminary motif classifications, by comparing each motif to the shared motifs and removing those that did not match. This process yielded the final motif clustering results (Table S1). Additionally, the types of motifs were further distinguished based on their representation in the shared motifs.

### 2.4. AlphaFold Interaction Structure Prediction

For each motif type, approximately one-quarter of the motifs were randomly selected for interaction structure prediction. For each selected motif, a protein whose DNA sequence information matched the motif was randomly chosen, and both the protein sequence information and corresponding DNA sequence information were input into the AlphaFold server [32] to predict the interaction structure between the dimeric protein and double-stranded DNA. Following the prediction, high-confidence results were selected for further experimental validation.

### 2.5. Protein Induction Expression

The protein expression process involved the *E. coli* BL21(DE3) strain, which was cultured in LB medium (1% tryptone, 0.5% yeast extract, 0.5% NaCl; agar added to 1.5% for solid medium) at 37 °C with kanamycin at a final concentration of 50 μg/mL as required. For the highly reliable protein structure prediction results selected, we extracted the corresponding coding DNA sequence and used it to construct the pET-28a expression vector. The expression vectors were synthesized by China Hangzhou Tsingke Biotechnology Co., Ltd. (see Table S3). After the vector synthesis was completed, each expression vector was separately introduced into *E. coli* BL21(DE3) cells by transformation and screened using kanamycin-containing agar plates. Colonies from these plates were picked and grown in LB liquid medium at 37 °C, shaking until the optical density at 600 nm (OD_600_) reached approximately 0.6. Subsequently, isopropyl β-D-1-thiogalactopyranoside (IPTG) was added to a final concentration of 0.2 mM, and the cultures were further incubated at 30 °C with shaking for 6 h to induce protein expression (the control was not supplemented with IPTG). After induction, the expressed proteins were analyzed using SDS-PAGE and Coomassie Blue staining to assess induction efficiency. Finally, the induced bacterial pellets were collected by centrifugation at 8000 rpm and stored at −80 °C for subsequent use.

### 2.6. Protein Purification

The bacterial pellets were resuspended in Buffer A (20 mM Tris-HCl, 5% glycerol, 1 M NaCl, pH 7.5) and lysed using an ultrasonic cell disruptor (2.5 s on, 7.5 s off, total duration of 40 min) on ice. The resulting suspension was centrifuged at 12,000 rpm for 30 min, and the supernatant was collected and filtered through a 0.22 μm sterile filter for subsequent use.

The protein purification system utilized was the AKTA pure 25 (GE Healthcare, Pittsburgh, PA, USA), equipped with a Ni column (HisTrap™ HP, 1 mL, Cytiva) and eluted using Buffer B (20 mM Tris-HCl, 5% glycerol, 1 M NaCl, 500 mM imidazole, pH 7.5). The nickel column was first equilibrated with Buffer A. After filtering the supernatant, the sample was loaded onto the nickel column. Once the column had re-equilibrated with Buffer A, impurities were washed away using 10% Buffer B. The target proteins were then eluted sequentially using 40% and 100% Buffer B.

The purified protein solution was analyzed by SDS-PAGE to confirm purity before being stored short-term at 4 °C or rapidly frozen with liquid nitrogen and stored at −80 °C for long-term preservation.

### 2.7. EMSA

The DNA samples used in the Electrophoretic Mobility Shift Assay (EMSA) are detailed in Supplementary Table S2. The procedure involves taking single-stranded DNA labeled with FAM at the 5′ end and mixing it with unlabeled complementary DNA at a ratio of 1:4. The final concentration of the labeled DNA is set to 1 μM. The annealing process begins by heating the mixture to 95 °C for 5 min, followed by a gradual cooling phase where the temperature decreases by 1 °C every minute until it reaches 4 °C. For the protein samples used in the EMSA, they are diluted using Buffer C (comprising 250 mM NaCl, 5% glycerol, and 20 mM Tris-HCl at pH 7.5) to achieve a concentration of approximately 10 μM. The reaction mixture is prepared by combining 2 μL of the DNA solution with 2 μL of the protein solution. The total volume is adjusted to 20 μL using Buffer C. This mixture is then incubated at 37 °C for 30 min. Following the incubation period, the samples are subjected to polyacrylamide gel electrophoresis (PAGE). The resulting gels are scanned and imaged using a Typhoon FLA 9500 system from GE Healthcare to analyze the electrophoretic patterns.

### 2.8. Genomic Motif Searching

For proteins interacting with DNA, the genomic sequence information of each protein was first identified: the DNA sequences upstream of each protein’s reading frame (200 bp) were extracted and concatenated into a FASTA-formatted file. Using known motifs as references, the fimo program was employed to search for motifs within the FASTA file (command: “nohup fimo --thresh 5e−5 --norc meme_out/meme.html promoter_seqs.txt”). Protein sequences successfully identified with motifs were collected. These sequences were then mapped through the EggNOG server, and the counts of each protein category were statistically analyzed.

## 3. Results

### 3.1. The Workflow and Result of Motif Discovery

**Figure 1:**
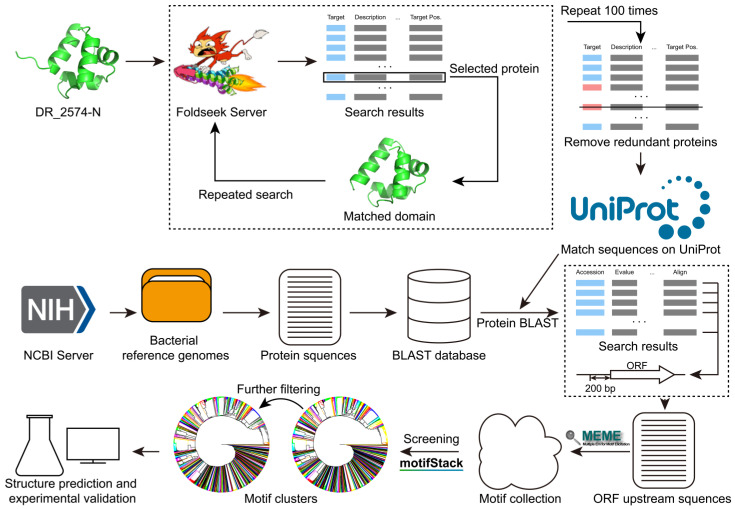
The workflow for collecting protein information and mining potential motifs. Initially, the N-terminal (XRE domain) of the predicted structure of DR_2574 was used as a template, and manual searches were conducted on the Foldseek server to identify proteins with XRE domain. Each search retrieved 1000 protein entries from the AlphaFold predicted structure database. From these entries, the last one that was structurally matching at XRE domain yet non-redundant with prior matches was selected, and its corresponding domain was extracted for further searches. This iterative process was repeated 100 times, with duplicates removed, resulting in the collection of protein entries with similar XRE domains. The bacterial reference genomes were downloaded from NCBI, and all protein sequences were extracted to build a local BLAST database. Subsequently, each collected protein entry was subjected to BLAST analysis, and the BLAST results were used to identify potential motifs upstream of the reading frames using the MEME suite. After preliminary screening, clustering was performed, and unreasonable clusters were removed. Finally, structural prediction and interaction experiments were conducted for verification.

**Figure 2:**
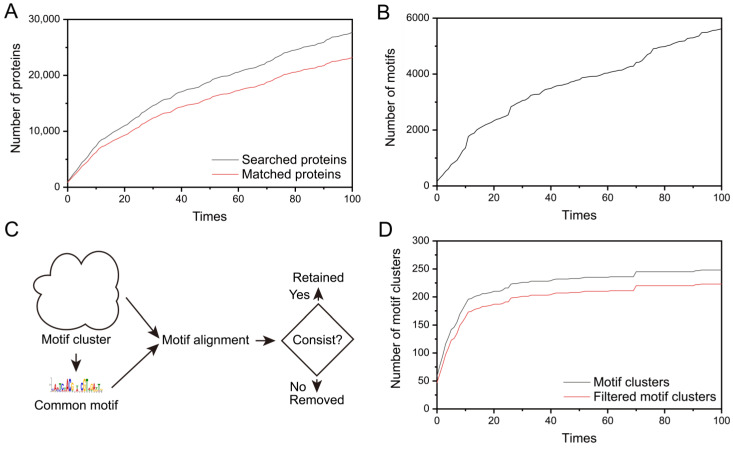
Data statistics related to protein collection and motif discovery during the process. (**A**) Statistics of protein collection from the Foldseek server with the number of collections. The black line represents the counts of de-redundant proteins, while the red line represents the counts after further matching against the Uniprot database. There is no significant difference in the trend between the two curves, and both still show an upward trend after 100 searches. (**B**) Number of motifs discovered with the number of collection times from the Foldseek server. After 100 searches, the upward trend of the curve remains obvious. (**C**) Workflow for further screening of motif clusters after motif clustering. For each motif cluster, common motifs among all members are firstly summarized. Then, the common motifs are compared with every individual motif in the cluster. If consist, the motif cluster is retained; otherwise, it is discarded. (**D**) Number of motif clusters with the number of collection times from the Foldseek server. The black line represents the initial number of motif clusters, while the red line represents the number after motif cluster screening. Notably, when the collection reaches around 70 times, there is no significant increase in the number of motif clusters.

The workflow for collecting protein information and mining potential motifs is roughly illustrated in Figure 1. Initially, using the N-terminal (XRE domain) of the predicted structure of DR_2574 as a template, we manually cycled through searches on the Foldseek server [31] to identify proteins with similar structures. Each search on Foldseek retrieved 1000 protein entries from the AlphaFold predicted structure database [32]. Among these protein entries, we continually selected the last entry that is structurally matching yet non-redundant with prior matches and extracted its corresponding domain for further searches, thereby repeating searching process. After repeating this process 100 times and removing duplicates, we ultimately gathered 27,732 protein entries with similar XRE domains (Figure 2A). We then conducted information and sequence matching of these protein entries in the UniprotKB [33], and retained 23,222 protein sequences (Figure 2A).

To discover the potential motifs upstream of these protein reading frames, we first downloaded the reference genomes from the bacterial domain from NCBI (National Center of Biotechnology Information) and extracted the protein sequences to construct a BLAST database. Subsequently, each of the 23,222 collected proteins was subjected to BLAST alignment. We extracted reading frame upstream sequences of each protein from the BLAST results and employed the MEME Suite [34] to identify potential motifs. By excluding proteins with insufficient BLAST matches and removing disordered sequences, we ultimately identified 5622 potential motifs (Figure 2B). Following this, we performed clustering analysis on the collected motifs using motifStack, initially grouping them into 248 clusters. Further refinement was conducted, and only clusters where all members shared identical core motifs were retained (Figure 2C), resulting in a total of 223 clusters (Figure 2D and Table S1).

### 3.2. Motif Analysis and Clustering

**Figure 3:**
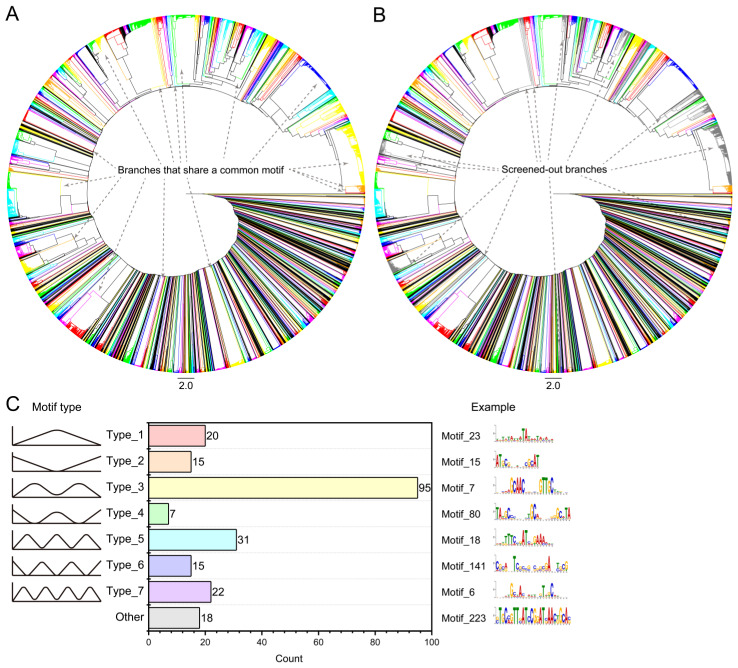
Results of motif discovery and clustering. (**A**) Clustering results after initial motif clustering. Each colored branch represents a motif cluster. The number of motif clusters obtained from the initial discovery is 248. (**B**) Results after screening of motif clusters. Colored branches represent retained clusters, while gray branches represent those removed. The number of motif clusters after screening is 223. (**C**) Classification results of motif clusters based on conserved base distribution patterns. Motif categories are divided into 7 classes and others, with Type_3 having the most entries.

In the process of discovering structurally similar proteins and hidden motifs, it can be observed that both the number of collected proteins and the number of scanned motifs consistently showed an upward trend (Figure 2A,B). Although a large number of motifs were collected, some exhibit consistent sequence patterns and can be categorized into the same motif. Therefore, given the large number of motifs obtained, we performed clustering on them and labeled the obvious motif clusters containing 5 and more motifs (Figure 3A). The clustering results indicated that there was no significant increase in the number of motif clusters (Figure 2D), when the collection reached approximately 70 times. Therefore, we speculated that the collection process up to 100 times has encompassed the main palindromic motifs of the XRE regulatory factors.

Subsequently, for each motif cluster obtained, we summarized the common motifs within them and compared these common motifs with every individual motif in the clusters. We screened out motif clusters with significant differences between common motifs and individual motifs, ultimately resulting in 223 motif clusters (Figure 2D, Figure 3B and Figure S1, Table S1). Notably, through our screening method, we also successfully identified the radiation and desiccation response motif (RDRM) corresponding to DdrO (Motif_81), which has been discovered by previous study [28].

Due to the significant sequence differences among these 223 motif clusters, to facilitate the differentiation of different motifs, we define the pattern where the central base is conserved while the flanking bases are not as a “peak”, and the pattern where the central base is not conserved while the flanking bases are as a “valley”. The distribution pattern of peaks and valleys across a motif can reflect the potential structural interaction modes between proteins and motifs. Based on these definitions, we classified motifs into 7 main types based on the base conservation patterns observed in motifs: Type_1 (single peak), Type_2 (single valley), Type_3 (double peak), Type_4 (double valley), Type_5 (triple peak), Type_6 (triple valley), and Type_7 (quadruple peak), as well as other indistinguishable categories (Figure 3C). The statistical results show that Type_3 (double peak, like “M” type) motifs are the most numerous, totaling 95 clusters, accounting for over 40% of all motif clusters. Additionally, RDRM is also categorized under Type_3.

### 3.3. Structure Prediction of Protein–DNA Motif Complex

**Figure 4:**
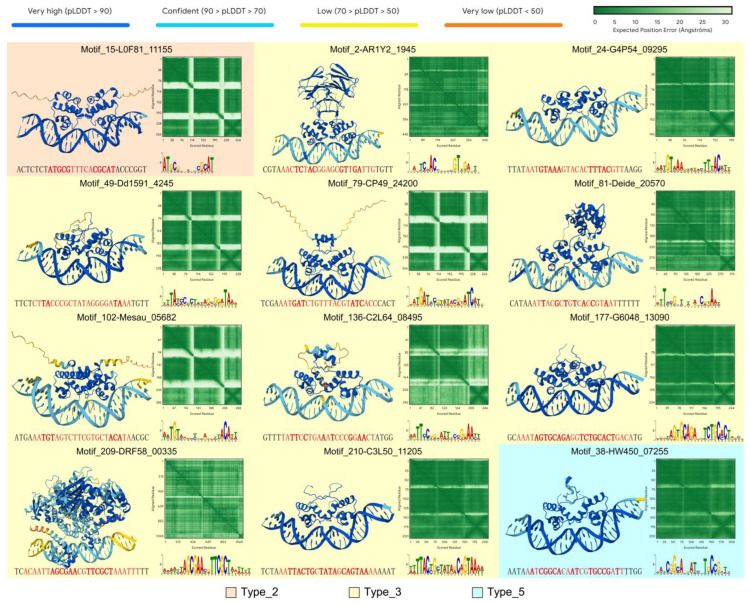
Structural predictions that demonstrate good interaction outcomes for protein–DNA interactions. In these prediction results, proteins and DNA exhibit good interaction patterns, and the HTH domain of proteins can form interaction interfaces with conserved bases in predicted motifs. In the DNA sequence, the red part represents the motif region, and the bold letters indicate the conserved bases.

To validate whether the collected XRE family protein can recognize and bind to the discovered motifs, we randomly selected approximately 1/4 of motifs from each type. For each selected motif cluster, we chose one representative protein and its corresponding motif-containing DNA sequence. By using AlphaFold server [32], we obtained the predicted interaction structures between the protein and double-stranded DNA (Figure 4). Notably, most well-predicted protein–DNA complex models exhibit a typical transcription factor binding mode, with two helices binding into the adjacent major grooves of the promoter DNA. The spacer length of the motifs appears around 12–14 bp, consistent with the distance of two major grooves, which also corresponds to the distance of the two helices. The variation in the spacer length may be explained by different XRE proteins and the bending angle of the promoter DNA.

In these predicted interaction structures, the reliability of protein–DNA interactions varies widely (Figure S2). Some structures show poor interaction reliability, or no interaction at all, such as Motif_175 and Motif_212 (Figure S2). Some structures have good interaction reliability but the interacting bases are not at our predicted conserved sites, such as Motif_23 and Motif_90 (Figure S2). We primarily screened based on higher interface predicted template modeling scores (ipTM), selecting samples where both ipTM and pTM (predicted template modeling score) are above 0.7 to include potentially correct structures for experimental validation. Additionally, we considered the predicted local distance difference test (pLDDT) values in the predicted interaction regions, as well as motifs indicating that the protein’s HTH domain can interact with conserved palindromic bases (Table S4). We believe that these criteria demonstrate high confidence and strong interaction strength. For example, with RDRM, the predicted structure of DdrO interacting with the RDRM site (Motif_81) has high reliability and shows a potential interaction between the HTH domain and conserved bases. We excluded those with low confidence from the predicted structures and selected those with high interaction structure reliability (Figure 4) for following validation.

### 3.4. Experimental Validation of Protein–DNA Interactions

Although we successfully scanned for potential motifs and predicted interaction structures using genomic data and bioinformatics tools like AlphaFold, these computational predictions are limited to in silico models, and their accuracy under real conditions requires experimental validation. As DdrO-RDRM interaction has been validated by previous studies [12,28,35], we proceeded to validate the binding affinity of the remaining 11 predicted models shown in Figure 4.

**Figure 5:**
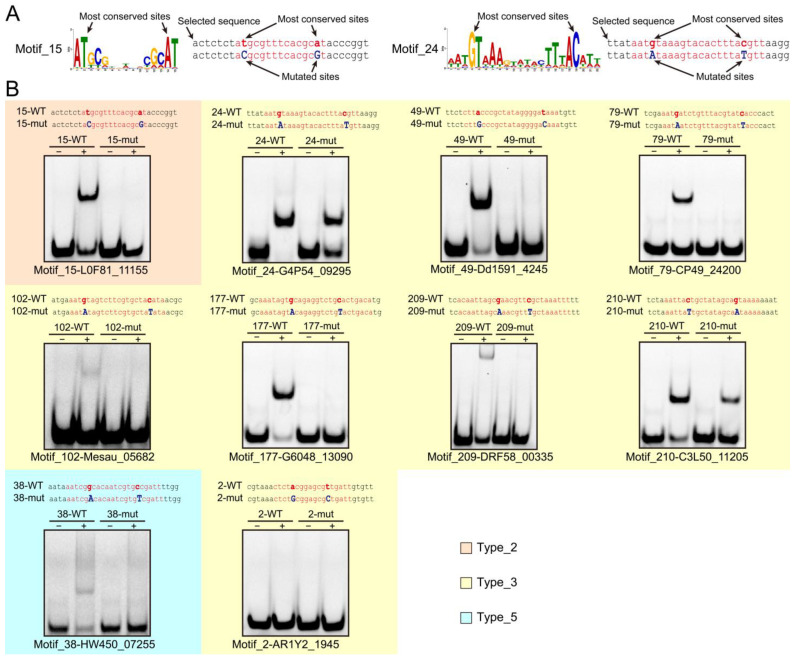
The experimental verification results of protein–DNA interactions. (**A**) Mutation indication of DNA sequence. Taking Motif_15 and Motif_24 as examples, we mutate the two most conserved nucleotides based on their motif patterns, following substitution rules: G ↔ A and C ↔ T. (**B**) EMSA results of protein–DNA interactions. Except for Motif_2, the proteins corresponding to the other 9 motifs successfully interacted with their respective double-stranded DNA. Furthermore, mutations in key bases weakened or disrupted these interactions, demonstrating the reliability of the predicted motifs. In the DNA sequence, the red indicate the motif regions, the bold letters represent the mutation sites, and the blue letters denote the bases after mutation.

We constructed expression vectors containing the genes of these 11 proteins and expressed target proteins using *E. coli* expression system. While the protein (C2L64_08495) corresponding to Motif_136 failed to express after being induced (Figure S3A), the other 10 proteins were successfully expressed and followed by purification (Figure S3B). After protein purification, since the most conserved sites may be involved in protein recognition and binding, we synthesized motif-containing DNA chains carrying 5′FAM fluorescence labels corresponding to each protein and introduced point mutations at the most conserved sites. Taking Motif_15 and Motif_24 as examples (Figure 5A), we selected the two most conserved nucleotides based on their specific motif forms and mutated them according to the rules of G-A and C-T. Following annealing of fluorescence labeled DNA chain with the complementary strand, we incubated the protein and the corresponding DNA substrate and analyzed their interactions using polyacrylamide gel electrophoresis (PAGE). PAGE results showed that, except for Motif_2, clear protein–DNA shift bands were observed in the other 9 groups. Mutations at conserved sites significantly weakened or abolished these bands, demonstrating the specific binding of predicted protein–motif pairs (Figure 5B). Additionally, the exceptional case of Motif_2 indicates that computational methods alone are insufficient to fully confirm the validity of predicted motifs, and experimental validation remains an indispensable verification approach.

Furthermore, we performed a cross-reactivity analysis using EMSA (Figure S4A–J). Based on the EMSA results, proteins typically exhibit the strongest binding to sequences containing the corresponding motif, except the Motif_2 protein (AR1Y2_1945) which showed no interaction with any of the 10 tested sequences (Figure S4A). Moreover, several transcription factors displayed cross-binding. For instance, the Motif_24 protein (G4P54_09295) can also interact with 79-WT and 102-WT (the fifth lane in Figure S4F,G). By comparing the sequence similarity between two sequences (79-WT and 210-WT) and Motif_24 (Figure S4K), it can be seen that the key bases of these sequences are consistently preserved, such as the most conserved base G. Similarly, the Motif_49 protein (Dd1591_4245) interacted with 210-WT (the seventh lane in Figure S4J), while Motif_210 protein (C3L50_11205) interacted with 49-WT (the last lane in Figure S4E). It can also be observed that 49-WT and 210-WT share a 5′-TTAC-3′ segment (Figure S4L), but the spacing between the corresponding segments is inconsistent. We hypothesize that the flexibility of both the DNA chain and the protein allows the protein to still interact with DNA. However, the intensity of the binding band after spacing modification is lower than that under the original spacing, which indicates that changes in spacing do indeed affect the protein–DNA interaction. Interestingly, when the sequences and motifs are quite different, some corresponding proteins can still exhibit binding capability. For example, Motif_210 protein (C3L50_11205) can still bind to both 38-WT and 102-WT (the last lane in Figure S4D,G), despite these sequences having little sequence similarity with Motif_210 (Figure S4M). The results show that corresponding protein may also have other binding sequences apart from the discovered motif.

## 4. Discussion

In the regulation of life metabolism, bacteria typically utilize specific transcription factors to mediate the transcription of related genes under different conditions. Among known families of transcription factors, the XRE family is a common one that participates in the transcriptional regulation of various life activities in bacteria. By summarizing the characteristics of XRE transcription factors, we found that these transcription factors typically exhibit autoregulation and often form dimers to bind promoter DNA, resulting in recognition motifs that usually present as palindromic forms.

Based on these two characteristics of XRE transcription elements, we extensively collected proteins carrying the HTH domain specific to the XRE family and scanned for potential palindromic motifs upstream of their reading frames. After completing the scanning and classification of motifs, we selected some motifs and used AlphaFold to predict protein–DNA interactions to verify the accuracy of our search results. Some predictions showed high reliability, and the following EMSA experiments confirmed the existence of most interactions. This indicates that our search and prediction process can help us to quickly identify potential XRE transcription factor recognition motifs with a certain degree of reliability.

Certainly, based on the results we collected, some palindromic motifs appear well-defined, but their predicted interaction structures do not show significant protein–DNA interactions. This situation occurs quite frequently. The binding of these proteins to DNA may require assistance from certain modifications or cofactors. It is also possible that these proteins have the HTH structure, but they do not actually perform the function of transcription factors. For example, there are proteins that contain not only the HTH domain but also a large part of other domains which may be involved in the actual function of the protein, such as Motif_178 in type 1, Motif_64 in type 2, Motif_204 in type 4, Motif_215 in type 5, Motif_212 in type 6, and Motif_6 in type 7 (Figure S2). Moreover, we speculate that some regular palindromic motifs may also be recognition sequences for other transcription factors, meaning that this transcription factor is also regulated by upstream proteins. Alternatively, the identified motifs could represent false positives generated by MEME clustering, or they might be accidental products of sequence randomness that lack any actual functional role.

Furthermore, experimental validation of predicted structures is crucial, since some well-predicted models failed to exhibit binding affinity. For example, Motif_2 showed a high degree of confidence in its predicted interactions, but no protein–DNA shift band was observed under experimental condition. Based on the cross-reactivity study, we found that the same transcription factor can bind to multiple sequences. The strength of this binding ability is typically related to the similarity between the sequences and corresponding motifs. Overall, most transcription factors exhibit more significant interactions with their own specific motifs (Figure S4).

Then, are transcriptional regulators whose interactions have been experimentally validated in this species solely involved in autoregulation? Typically, transcription factors bind to corresponding motif sequences to regulate genes. For instance, the transcription repressor DdrO in *Deinococcus* binds to the promoters of DNA damage response (DDR) genes at RDRM sites [36]. The *Staphylococcus pathogenicity* islands (SaPIs) are regulated by repressor ImmR, which typically binds to the promoter region of functional genes [37]. We scanned and statistically analyzed the distribution of reading frames for verified palindromic motifs in corresponding species (Figure S5). It can be observed that genes carrying the RDRM (Motif_81) are concentrated in recombination and repair functional categories, consistent with RDRM’s role in managing DNA damage repair. Some motifs (like Motif_15, 38, 49, 79, 177) are concentrated at the upstream sequences of transcription-related genes and may regulate these genes. Notably, Motif_49 also appears upstream of genes related to amino acid transport and metabolism; we speculate that Motif_49 may be involved in the uptake and utilization of amino acids by bacteria. Other motifs are either associated with genes of unknown function (e.g., Motif_24, 209, 210) or are sparsely distributed in the genome (Motif_102). Of course, the specific regulatory networks involving these transcriptional regulators and motifs still require further experimental validation.

However, our method is limited to widespread transcriptional regulators that interact with DNA in dimeric forms. Some functional transcriptional regulators with narrow distribution are difficult to detect. Additionally, some transcriptional regulators may interact with target DNA sequences as monomers or polymers and might require other auxiliary proteins to bind DNA. In such cases, the motifs they recognize may be no longer palindromic. Certainly, based on the EMSA results, some proteins may have multiple binding motifs, while our method is not sufficient to identify all the motifs of a single regulator. Additionally, the identified motifs may also belong to upstream regulators, be clusters of non-functional sequences, or result from overprediction by computational methods. Therefore, computational results cannot replace experimental validation.

## 5. Conclusions

In summary, by collecting homologous proteins and scanning upstream sequences of their reading frames, we could identify potential transcriptional regulator recognition motifs. Through clustering analysis, we determined widespread motif types and validated the interaction between some transcriptional regulators and motifs using structural prediction and experimental validation. This provides a reliable reference for subsequent studies on metabolic pathway regulation in bacteria, and contributes to advancing our systematic understanding of bacterial regulatory networks. Furthermore, the method does not rely on the XRE regulators themselves, but rather on two characteristics: autoregulation and palindromic sequences. Therefore, if transcription factors from other families clearly possess these two characteristics, our method can identify the potential motifs of these regulators. This will further facilitate the study of bacterial metabolic regulatory pathways and provide potential support for fields such as the utilization of beneficial microorganisms and the prevention and control of pathogenic microorganisms.

## Data Availability

The raw data supporting the conclusions of this article will be made available by the authors on request.

## Supplementary Materials

The following supporting information can be downloaded at: https://www.mdpi.com/article/10.3390/life15101577/s1, Figure S1: Results of motif screening; Figure S2: Results of the interaction structure prediction; Figure S3: Experimental validation of protein expression and purification of selected proteins; Figure S4: Cross-reactivity analysis in protein-DNA interactions; Figure S5: Distribution of motifs within the corresponding species; Table S1: The motif clusters discovered in this study; Table S2: Primers and DNA substrates; Table S3: The plasmids used in this study; Table S4: Structure Prediction of Protein-DNA Motif in this study.
