# Supplementary material for: Computational–Experimental Identification of Palindromic Motifs Bound by Bacterial XRE Family Transcriptional Regulators
Source: Life (Basel). 2025 Oct 10;15(10):1577. doi: 10.3390/life15101577 (PMC12565409; doi:10.3390/life15101577)
Supplement: Supplementary file 1 [file life-15-01577-s001.zip › life-3880253-supplementary.pdf]

Supplementary data

Computational-experimental identification of palindromic motifs bound by bacterial XRE family transcriptional regulators

Linjia Wang[1,#], Shitong Zhong[1,#], Liangyan Wang[1], Huizhi Lu[1]* & Yuejin Hua[1,2]*

[1.]MOE Key Laboratory of Biosystems Homeostasis & Protection, Institute of Biophysics, College of Life Sciences, Zhejiang University, China

[2.] Cancer Center, Zhejiang University, Hangzhou, Zhejiang, China

[#.] These authors contributed equally to this work.

* To whom correspondence should be addressed. Tel: 86-571-86971703; Fax: 86-571-86971703; Email: yjhua@zju.edu.cn

Correspondence may also be addressed to huizhilu@zju.edu.cn

Keywords: palindromic motif, XRE Family, transcriptional regulators, motif clustering, AlphaFold, protein-DNA interactions

Supplementary Figures

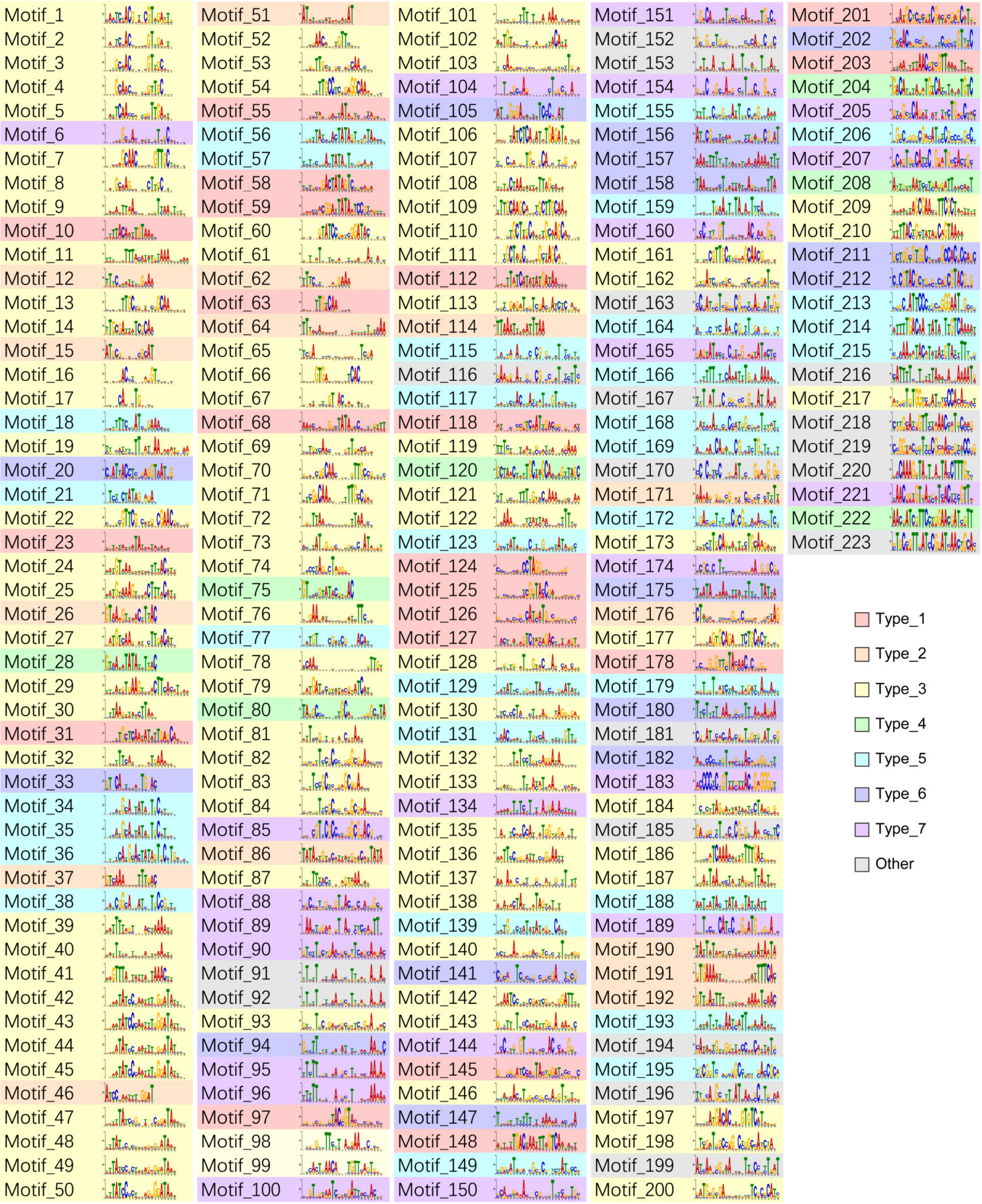

Supplementary Figure S1: Results of motif screening. A total of 223 motif clusters were identified through our screening method. These clusters were subsequently classified into 7 main types and indistinguishable category based on the pattern of conserved bases in motifs.

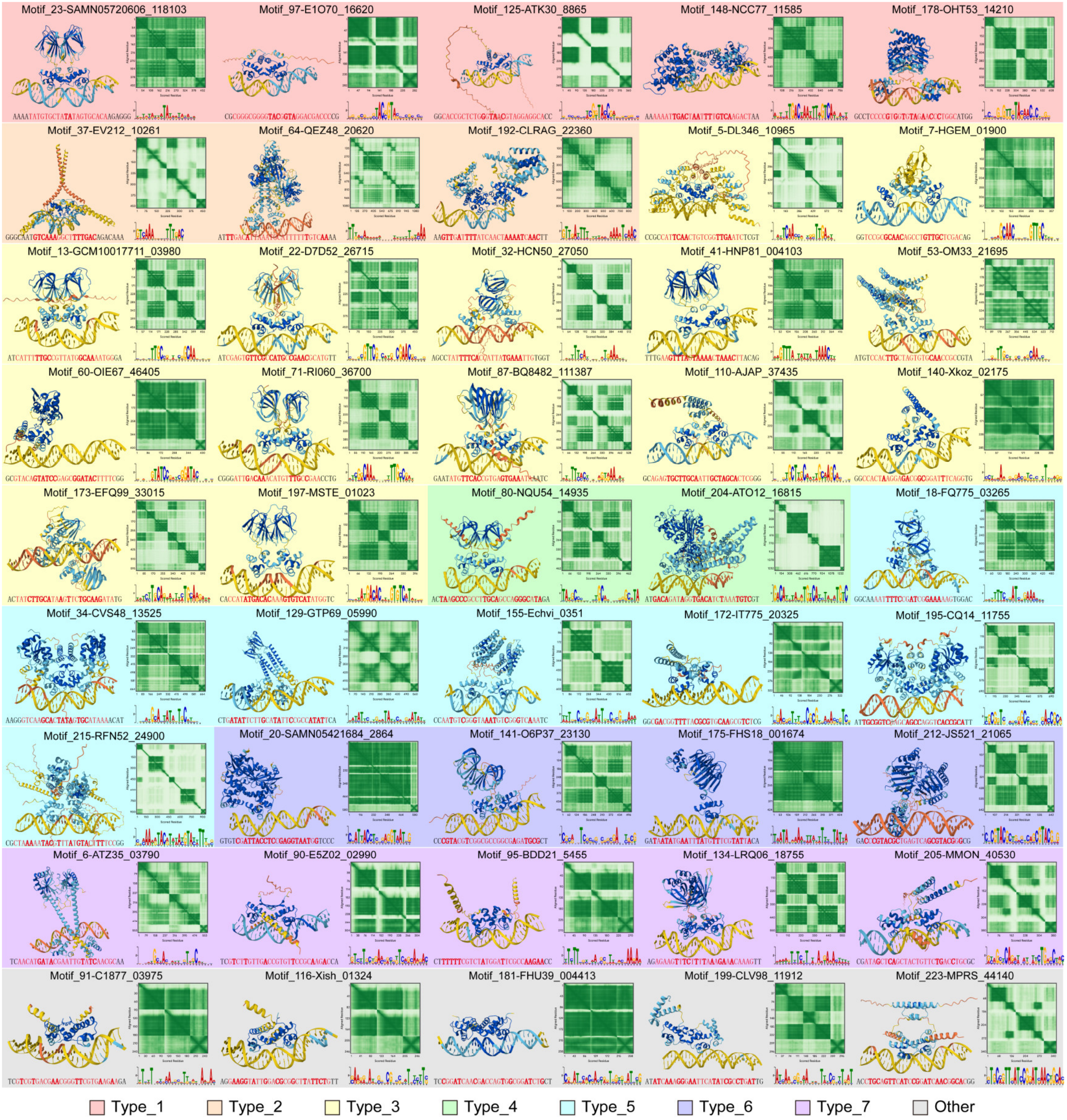

Supplementary Figure S2: Results of the interaction structure prediction. For each type of motif cluster, approximately one-fourth is selected for use in the results of protein-DNA interaction structure prediction. The well-performing interaction structure results are displayed in Figure 4.

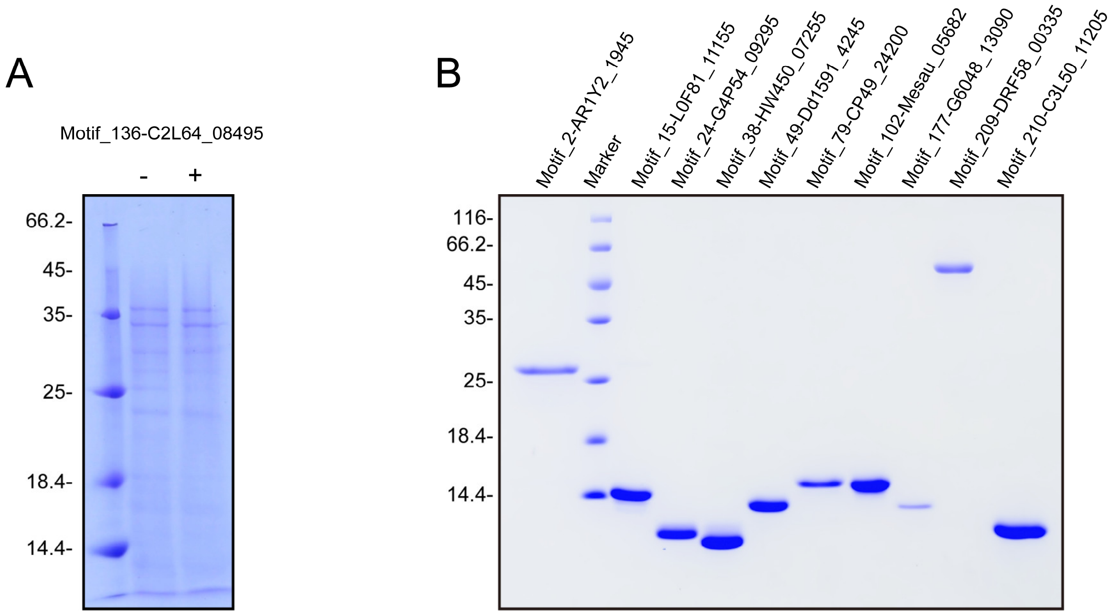

Supplementary Figure S3: Experimental validation of protein expression and purification of selected proteins. (A) The induction of the protein corresponding to motif_136 was unsuccessful. "-" represents uninduced cell lysate, and "+" represents induced cell lysate. (B) The SDS-PAGE results after purification of the remaining 10 proteins.

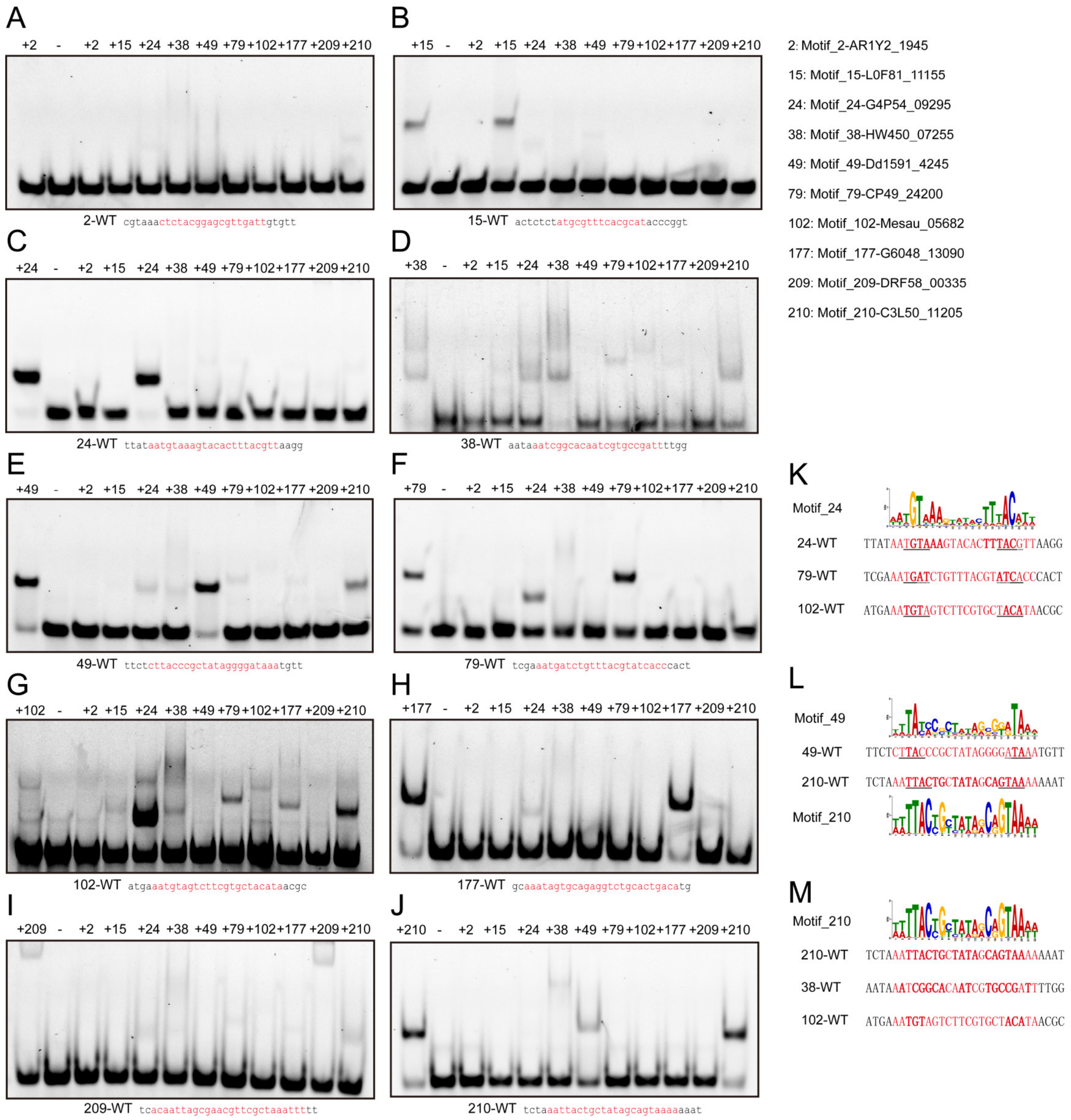

Supplementary Figure S4: Cross-reactivity analysis in protein-DNA interactions. (A-J) The EMSA results after protein-DNA cross incubation. Some proteins showed binding activity to other sequences, such as Motif_24 protein (G4P54_09295), Motif_49 protein (Dd1591_4245), and Motif_210 protein (C3L50_11205). From the results, it is generally observed that proteins exhibit the strongest binding affinity to sequences containing their corresponding motifs. (K) Comparison of similarity between Motif_24 and some sequences (79-WT, 102-WT). (L) Comparison of similarity between Motif_49 and Motif_210. (M) Comparison of similarity between Motif_210 and some sequences (38-WT, 102-WT).

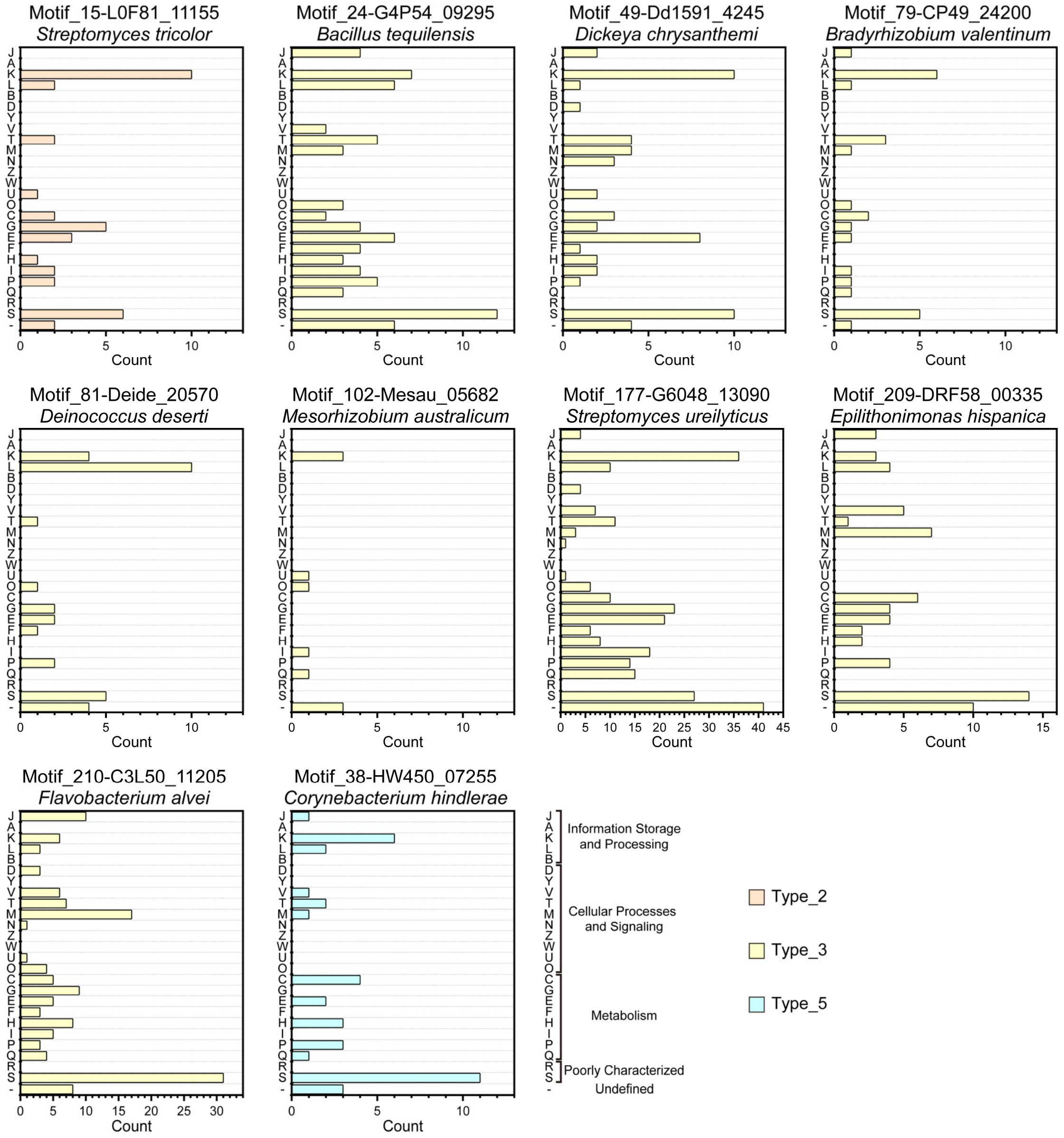

Supplementary Figure S5: Distribution of motifs within the corresponding species. The distribution of motifs that have been successfully validated in protein-DNA interaction experiments across various signaling pathways in the corresponding species. One letter code descriptions: [J] Translation, ribosomal structure and biogenesis; [A] RNA processing and modification; [K] Transcription; [L] Replication, recombination and repair; [B] Chromatin structure and dynamics; [D] Cell cycle control, cell division, chromosome partitioning; [Y] Nuclear structure; [V] Defense mechanisms; [T] Signal transduction mechanisms; [M] Cell wall/membrane/envelope biogenesis; [N] Cell motility; [Z] Cytoskeleton; [W] Extracellular structures; [U] Intracellular trafficking, secretion, and vesicular transport; [O] Posttranslational modification, protein turnover, chaperones; [C] Energy production and conversion; [G] Carbohydrate transport and metabolism; [E] Amino acid transport and metabolism; [F] Nucleotide transport and metabolism; [H] Coenzyme transport and metabolism; [I] Lipid transport and metabolism; [P] Inorganic ion transport and metabolism; [Q] Secondary metabolites biosynthesis, transport and catabolism; [R] General function prediction only; [S] Function unknown; [-] Undefined.
